# Lipid Metabolism Disorders in the Comorbid Course of Nonalcoholic Fatty Liver Disease and Chronic Obstructive Pulmonary Disease

**DOI:** 10.3390/cells10112978

**Published:** 2021-11-01

**Authors:** Stanislav Kotlyarov, Aleksei Bulgakov

**Affiliations:** Department of Nursing, Ryazan State Medical University, 390026 Ryazan, Russia; bulgakov.alexey92@gmail.com

**Keywords:** non-alcoholic fatty liver disease, lipid metabolism, metabolism, chronic obstructive pulmonary disease

## Abstract

Non-alcoholic fatty liver disease (NAFLD) is currently among the most common liver diseases. Unfavorable data on the epidemiology of metabolic syndrome and obesity have increased the attention of clinicians and researchers to the problem of NAFLD. The research results allow us to emphasize the systemicity and multifactoriality of the pathogenesis of liver parenchyma lesion. At the same time, many aspects of its classification, etiology, and pathogenesis remain controversial. Local and systemic metabolic disorders are also a part of the pathogenesis of chronic obstructive pulmonary disease and can influence its course. The present article analyzes the metabolic pathways mediating the links of impaired lipid metabolism in NAFLD and chronic obstructive pulmonary disease (COPD). Free fatty acids, cholesterol, and ceramides are involved in key metabolic and inflammatory pathways underlying the pathogenesis of both diseases. Moreover, inflammation and lipid metabolism demonstrate close links in the comorbid course of NAFLD and COPD.

## 1. Introduction

Metabolic disorders are one of the most important problems of modern society [1]. Metabolic syndrome and related diseases are an important part of the epidemiology of chronic non-communicable diseases. Their prevalence is growing in many countries of the world, increasing the economic and social burdens [2,3].

Non-alcoholic fatty liver disease (NAFLD) is closely associated with impaired metabolic processes and is considered one of the most common liver diseases in European countries. Its prevalence among the adult population varies depending on the method of diagnosis, age, sex, and some other characteristics and, according to various data, ranges from 17% to 46% [4]. Such data roughly correspond to the occurrence of metabolic syndrome, which increases the risk of developing a severe form of the disease. NAFLD confidently demonstrates negative growth trends in parallel with the prevalence of obesity and type 2 diabetes, the key problems of modern society associated with impaired metabolism [5]. It is assumed that the increase in the prevalence of NAFLD in the coming years will make it one of the leading causes of liver diseases, requiring its transplantation [6,7,8,9]. It is important to note that NAFLD occurs not only in overweight and obese people, but also in 7% of people with normal body weight, often in young women with normal levels of liver enzymes [10,11]. These data, as well as information about excessive health care costs associated with the disease, demonstrate the importance of NAFLD as a serious health problem worldwide [12].

Non-alcoholic fatty liver disease (NAFLD) is characterized by excessive accumulation of fat in the liver and is determined in the presence of steatosis in more than 5% of hepatocytes according to the results of histological examination or with a proton density of the fat fraction >5.6% according to proton magnetic resonance spectroscopy (PMRS) or fat–water MRI (magnetic resonance imaging). NAFLD includes two morphological forms with different prognosis: nonalcoholic fatty liver (NAFL) and non-alcoholic steatohepatitis (NASH). The severity of the disease in NASH is very variable, including fibrosis, the activity of which correlates with the stage of the disease, and cirrhosis. A liver biopsy should be performed to establish the final diagnosis of NAFLD.

The diagnosis of NAFLD involves the exclusion of secondary causes and significant alcohol consumption (more than 30 g per day for men and 20 g per day for women) [13]. Alcohol consumption in doses exceeding those specified indicates alcoholic liver disease. It should be noted that a moderate amount of alcohol can also contribute to the development of NAFLD in patients with metabolic risk factors. At the same time, the overall effect of metabolic risk factors on the development of steatosis exceeds the effect of alcohol in these patients [14].

Given the close and incompletely understood association of NAFLD with impaired metabolism, as well as the lack of understanding of all mechanisms of pathomorphological and clinical heterogeneity of the disease, another term, which is believed to better reflect current understanding of the pathogenesis of this disease, metabolically associated fatty liver disease (MAFLD), was proposed in 2020 [15,16]. The use of this term, as suggested by experts, will allow leveling out the influence of other liver diseases and alcohol intake in the diagnosis, while emphasizing the heterogeneity of all fatty liver diseases, and can also help in stratifying patients in order to choose the best treatment strategy [16,17]. It should be noted that discussions regarding terminology continue, which once again underlines the complexity of the problem and the need for a better comprehensive analysis of it.

The relevance of the NAFLD problem is also due to the fact that patients may not seek medical care for a long time, and reliable tools required to confirm the diagnosis are not available to clinicians or are characterized by high cost and require qualified personnel, which is a serious problem for many countries of the world. Such conditions reduce the possibility of early diagnosis and prompt choice of an effective therapeutic intervention. In addition, NAFLD patients are more likely to have comorbidities that can present a much more severe clinical picture and are a major reason for seeking medical care. Among the most significant diseases that are associated with NAFLD are cardiovascular diseases [17]. This relationship is well demonstrated by the commonality of key risk factors and some links of pathogenesis.

**Figure 1:**
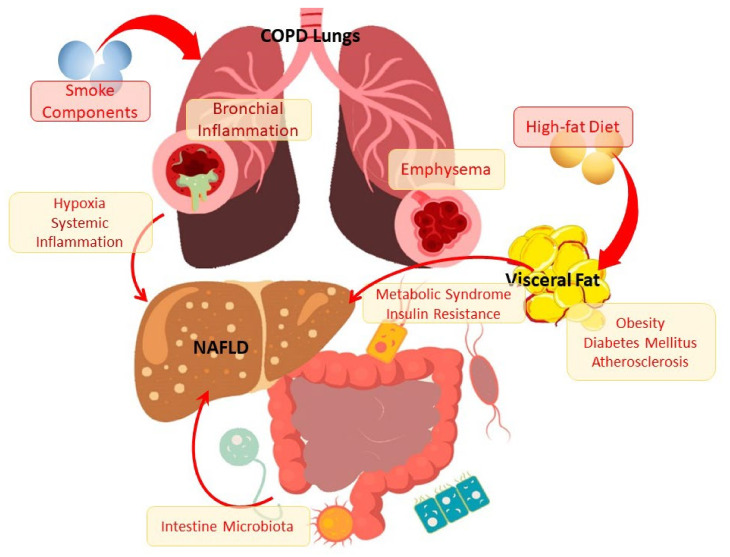
The scheme of the relationship between NAFLD and COPD through metabolic disorders and inflammation.

It is of interest to know that there is a higher prevalence of NAFLD among patients with chronic obstructive pulmonary disease (COPD). It was found that the frequency of steatosis, NASH, and fibrosis in COPD patients is 41.4%, 36.9%, and 61.3%, respectively [18,19]. However, the data on the mechanisms of disease communication are incomplete and partially contradictory, which can be explained by the difficulties of diagnosis and variability of both NAFLD and the clinical heterogeneity of COPD. These connections are probably complex and include the involvement of impaired metabolic mechanisms, inflammation, and the resulting effects on organs and tissues of decreased pulmonary function and hypoxia (Figure 1). It is shown that the severity of NAFLD is characterized by an independent association with a decrease in pulmonary function [20]. At the same time, a greater decrease in forced expiratory volume in 1 s (FEV1) corresponds to a greater degree of liver steatosis [21]. Patients with COPD and NAFLD with severe liver fibrosis showed a higher risk of cardiovascular events than in the general population of patients with liver fibrosis [19].

The purpose of this review was to discuss the role of impaired lipid metabolism as a link in the complex chain of processes underlying the development of NAFLD and COPD and their comorbid course.

## 2. Clinical, Morphological, and Pathogenetic Characteristics of NAFLD

As already noted, NAFLD shows a close bidirectional relationship with the metabolic syndrome [22]. The development of a metabolic syndrome can not only precede NAFLD, but also be its consequence [23,24]. Indeed, NAFLD almost doubles the risk of developing metabolic syndrome in the next few years [18,24]. In addition, studies show that the presence of NAFLD can be considered as an independent risk factor for a number of cardiovascular diseases [25,26,27,28]. Given that NAFLD is often combined with metabolic diseases, such as obesity, type 2 diabetes, hyperlipidemia, and hypertension, such comorbidity can lead to a significant increase in the risk of mortality [6,29,30].

A key characteristic of NAFLD, highlighting the association of the disease with the metabolic syndrome, is insulin resistance, which may have several mechanisms of development [31,32]. Insulin resistance not only mediates further impairment of metabolic parameters, but may also increase inflammation in NASH, as evidenced by the frequently more severe histopathological changes in the liver in patients with type 2 diabetes [32,33].

Thus, taking into account these data, the typical phenotype of a patient with NAFLD is an overweight person. Indeed, body mass index (BMI) and NAFLD show a strong correlation. However, some data indicate the presence of NAFLD in non-obese individuals [34,35,36]. Moreover, although most of these data were obtained in Asian countries, they were described all over the world [37,38,39,40,41,42,43,44,45,46,47]. Histological differences in NAFLD in obese and non-obese patients are minimal and consist of a lower degree of steatosis in people with NAFLD without obesity and a higher degree of fibrosis in NAFLD without obesity [48].

This information allows us to expand our views on the essence of metabolic processes in NAFLD, as well as comorbid relationships of NAFLD in obese and non-obese patients. It has been shown that individuals with NAFLD demonstrate a high risk of coronary atherosclerosis, regardless of the presence or absence of obesity [49]. There is evidence that patients with NAFLD and underweight may have a higher risk of developing diabetes and cardiovascular diseases than overweight people without NAFLD [50].

Despite numerous fundamental and clinical studies, the detailed pathogenetic mechanism of NAFLD is still unclear. It is believed that an important place in the pathophysiology of NAFLD is occupied by systemic and hepatic insulin resistance, de novo lipogenesis, disorders in the structure of the intestinal microbiota, local and systemic inflammation, and oxidative stress [51,52].

The classical theory of the pathogenesis of NAFLD assumed to a certain extent a step-by-step effect on the list, which is why it was called the “two hits” theory [53]. It was assumed that the first “hit” is associated with a negative effect on the liver of obesity, type 2 diabetes, dyslipidemia, and other metabolic risk factors and leads to hepatic steatosis. The second “hit” is associated with the action of oxidative stress and pro-inflammatory cytokines and leads to damage to the hepatocellular system and inflammation of the liver [54]. It has been shown that a three-day feeding of animals with food with a high-fat content provokes the accumulation of lipids in the liver and the development of insulin resistance in the liver. It is noteworthy that these changes took place without a significant increase in the mass of visceral fat or the concentration of fatty acids in the portal vein [55].

In recent years, the “multiple parallel hits” model has been proposed as a new model of the pathogenesis of NAFLD, since many factors can act in parallel, which ultimately leads to liver inflammation [55,56]. According to this concept, multiple damaging effects, the source of which is primarily the intestines and adipose tissue, can contribute to liver inflammation [52,57].

Indeed, the research data convincingly demonstrate the correlation of the amount of visceral fat with the degree of hepatic steatosis and inflammation. This correlates well with the information that pro-inflammatory changes in adipose tissue are a frequent phenomenon in pathological obesity, and this tissue may reflect the main source of cytokines that can attack the liver, thereby contributing to its inflammation [55].

## 3. Disorders of Lipid Metabolism in the Development and Progression of NAFLD

The key histological characteristic of NAFLD is the cellular accumulation of lipid droplets containing triacylglycerols (TAG), in the metabolism of which the liver plays an active role [58,59]. Lipid droplets range from 50 nm to 1 micron in diameter and have a unique structure that includes a hydrophobic core consisting of neutral lipids and a monolayer phospholipid membrane, as well as proteins that perform structural and functional roles [60,61,62].

In addition to accumulation in lipid droplets, TAG can be packed into very-low-density lipoproteins (VLDL), which are secreted from the cell [63]. An increase in VLDL secretion due to a high lipid content in the liver is the cause of complex dyslipidemia associated with NAFLD. It is interesting that a decrease in VLDL production, on the one hand, reduces the severity of dyslipidemia, but at the same time exacerbates the course of NAFLD, which emphasizes the complexity of the observed cross-links.

The currently most common model for lipid droplet formation suggests that excess TAG accumulates on the membranes of the endoplasmic reticulum, forming lipid bilayers of TAG, cholesterol esters in the center, and a monolayer of phospholipids and sphingomyelin outside [62,64,65,66]. The biosynthesis of TAG is closely related to the entry into the cell of free fatty acids coming from food or the release from adipose tissue cells [60,67]. Additionally, vice versa, TAGs from lipid droplets are a source of free fatty acids, which are an important part of the way to provide the cell with energy in the form of adenosine triphosphate (ATP). In addition to the energy function, lipid droplets serve as nodes for the exchange of fatty acids required for participation in other cellular processes [62].

### 3.1. Role of Free Fatty Acids

Excessive accumulation of lipid droplets in hepatocytes is a sign of steatosis, a key pathological process in non-alcoholic fatty liver disease, obesity, insulin resistance, and metabolic syndrome, which is considered as an imbalance between the process of storing and using lipids [58,60,63,68]. However, experimental data indicate that the accumulation of TAG in the liver alone is not enough for the development of insulin resistance [69]. Moreover, there is currently extensive data indicating that the accumulation of TAG in itself is not harmful to hepatocytes and can even be considered as a protective mechanism against lipotoxicity induced by free fatty acids [70,71,72]. This is supported by the fact that an excess of free fatty acids in fat cells can induce processes leading to cell dysfunction (lipotoxicity) and apoptotic cell death (lipoapoptosis) [71,73,74].

Indeed, a high level of free fatty acids in the blood serum demonstrates correlations with NAFLD and can be considered as a marker of progressive fibrosis [75]. To the greatest extent, these high levels are due to oleic and palmitic acids, which make up the main share of circulating free fatty acids [76].

It is interesting that it is not only the quantitative values of the level of free fatty acids that matter. An important indicator of hepatocyte damage can be the ratio of exogenous monounsaturated fatty acids and saturated fatty acids (SFA). In experiments, the accumulation of unsaturated fatty acids led to a significant increase in the content of TAG but was not accompanied by a decrease in cell viability, while excessive accumulation of saturated fatty acids in the liver contributed to the development of hepatocellular apoptosis, liver damage, and steatohepatitis [71,73,77].

The molecular mechanisms of this phenomenon may be based on changes in the activity of the enzyme steroyl-CoA-desaturase-1 (SCD1), whose deficiency leads to impaired liver adaptive mechanisms to the intake of excess saturated fatty acids and the development of steatohepatitis and fibrosis [78,79].

Excessive intake of free fatty acids into hepatocytes overloaded with lipids contributes to the overload of mitochondria and activates apoptotic mechanisms in the cell.

**Figure 2:**
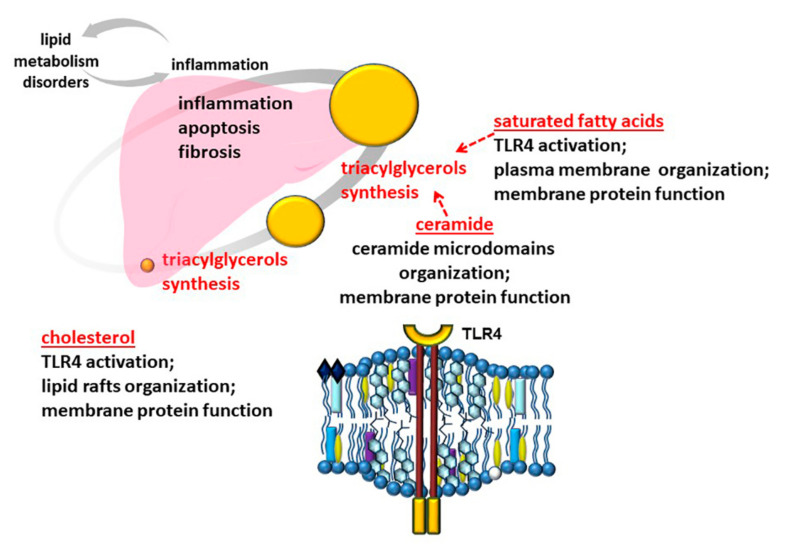
The scheme of lipid participation in the mechanisms of NAFLD development. Ceramides, cholesterol, and SFA demonstrate a link to inflammation through the regulation of cell plasma membrane structure and function and are involved in TAG formation and liver fibrosis.

Free fatty acids are an important link mediating the relationship between lipid metabolism and inflammation [80,81,82]. SFA may be involved in the activation of the receptor of innate immunity Toll-like receptor 4 (TLR4) (Figure 2) [83,84,85]. This action can be realized both through the incorporation of fatty acids into the phospholipids of the plasma membrane and the influence on their biophysical properties, and through direct stimulation of the receptor, demonstrating an evolutionary link to the lipopolysaccharide structure of Gram-negative bacteria, which the receptor is aimed at detecting [84]. Moreover, unsaturated fatty acids do not possess the ability to stimulate TLR4 and their effect on the biophysical properties of plasma membranes is opposite to [84]. It is interesting that intermittent hypoxia, which is a frequent result of obstructive sleep apnea, in a mouse model of diet-induced obesity leads to liver fibrosis via TLR4 receptor signaling pathways [86]. Tobacco smoke can also stimulate TLR4, realizing its proinflammatory effect.

In parallel, resident liver macrophages, the Kupffer cells, uptake large amounts of free fatty acids, which contribute to their proinflammatory activation. They produce proinflammatory cytokines, including interleukin (IL)-6 and tumor necrosis factor (TNF)-α, which are considered to be participants in the development of NASH.

Hepatocytes exposed to apoptosis form apoptotic bodies that are phagocytosed by hepatic stellate cells (HSCs) and Kupffer cells, which trigger a profibrogenic response due to transdifferentiation of HSCs into collagen-producing myofibroblasts. Accumulation of collagen is accompanied by an increase in metalloproteinases such as MMP-9. In addition, a study in mice found increased activity of tissue inhibitor of metalloproteinase 1 (TIMP-1) in fibrous tissue, which shifts the balance toward synthesis of extracellular matrix products. This, as well as exposure to other bioactive molecules such as alpha-2 macroglobulin (A2M), tilts the balance towards fibrogenesis, which is a key link in the development of liver fibrosis in NAFLD [87].

It is interesting that the severity of fibrosis in the liver may have a prognostic value in patients with COPD, as it is independently associated with an increased risk of COPD phenotype with frequent exacerbations [88].

Thus, although the most striking histological feature of NAFLD at all stages is the accumulation of TAG, the significance of this phenomenon in the pathogenesis of NAFLD is currently controversial. These lipids are believed to have no biologically relevant activity and are merely inert markers of the condition. Instead, studies have revealed the role of free fatty acids and sphingolipids, such as ceramides, in each stage of NAFLD: fat accumulation, insulin resistance, mitochondrial dysfunction, apoptosis, and fibrosis [58].

Given the importance of fatty acids in the pathogenesis of NAFLD, it is of interest to learn about the role of Peroxisome proliferator-activated receptors (PPARs) and their related therapeutic potential in NAFLD [89]. The PPAR family is known to include three isotypes, which are designated PPAR-α, PPAR-δ (-β), and PPAR-γ [89,90,91]. While PPAR-α is mainly expressed in the liver, PPAR-γ is mainly expressed in white adipose tissue as well as macrophages and Kupffer cells [92]. Their function is closely related to lipid metabolism. PPAR-α promotes fatty acid import into the mitochondria and β-oxidation in the mitochondria by activating Carnitine palmitoyltransferase 1A (CPT1A), acyl-CoA dehydrogenase medium chain (ACADM), and acyl-CoA dehydrogenase very long chain (ACADVL) [92]. PPARa is less pronounced in the liver of patients with NAFLD, so some PPARa agonists are being considered as potential drugs for treatment.

### 3.2. Role of Cholesterol

Known evidence suggests the involvement of cholesterol homeostasis disorders in the pathogenesis of steatohepatitis [93,94,95,96]. Cholesterol can modulate inflammation in the liver and, therefore, contribute to the progression of the disease. Dietary cholesterol has been shown to significantly increase the risk of cirrhosis or liver cancer, but the risk of developing these diseases was not correlated with serum cholesterol levels [96].

Epidemiological data show that increased cholesterol intake contributes to the risk of the development and severity of NAFLD. At the same time, the accumulation of free cholesterol in the liver increases the risk of NASH and fibrosis in NAFLD patients [94,95,97]. Free cholesterol causes hepatocyte apoptosis and necrosis by activating c-Jun *N*-terminal kinase 1 (JNK1) [98]. The increased intracellular concentration of free cholesterol in hepatocytes leads to its crystallization, and the dead hepatocytes containing cholesterol crystals initiate the process of Kupffer cells’ aggregation into “crown-like structures” that engulf dead cells, transforming into activated foam cells [96,99]. Such activation of Kupffer cells entails activation of stellate cells of the liver through the release of proinflammatory cytokines, which has a profibrogenic effect. Free cholesterol accumulating directly in HSCs also aggravates hepatic fibrosis [100,101,102].

Accumulation of free cholesterol in NAFLD may be associated with an imbalance among its entry into the cell, formation, and export from the cell. It is worth noting that NAFLD/NASH patients show increased expression of Sterol regulatory element-binding protein 2 (SREBP-2) transcription factor, which controls cholesterol homeostasis. Hyperinsulinemia associated with concomitant NAFLD insulin resistance and inflammatory stress contributes to this effect [103]. The combination of all these factors in NASH may be responsible for the persistent activation of SREBP2, bypassing the inhibitory effect of high cellular cholesterol levels that develop through a negative feedback mechanism [103,104].

In addition to increased cholesterol synthesis, changes in cholesterol excretion pathways have also been noted in patients with NASH. Decreased expression of Cholesterol 7 alpha-hydroxylase (CYP7A1) has been reported in NAFLD patients, resulting in decreased synthesis of bile acids from cholesterol [105]. A decrease in the expression of cholesterol transporters ABCG5/G8, responsible for the excretion of cholesterol with bile, has also been reported [106]. Thus, it is clear that the accumulation of free cholesterol in the liver in patients with NAFLD/NASH is multifactorial, based on increased cholesterol synthesis and decreased cholesterol elimination.

Some researchers suggest that another member of the ABC transporter family, ABCA1 [106,107], may also play a role in the pathogenesis of NAFLD. The functions of ABCA1 are well known for its role in reverse cholesterol transport, a process that ensures the export of cholesterol from the cell to an extracellular acceptor. Because of this, the transporter provides not only the regulation of cholesterol content in the cell but can also influence some of its functions. This is associated with the role of cholesterol in inflammation and in the organization of the lipid bilayer of plasma membranes. Excess cellular cholesterol content leads to the formation of oxysterols, which stimulate ABCA1 expression via LXR (liver X receptor) [108,109,110,111].

High-density lipoproteins (HDLs) carry out the transport of cholesterol from peripheral tissues to the liver, exerting an antiatherogenic effect. ABCA1 is involved in the formation of HDL, which determined its leading role in the pathogenesis of atherosclerosis [112,113]. These findings are based on the results of many studies conducted in patients with dyslipidemia, coronary heart disease, and atherosclerosis. *ABCA1* gene polymorphism has also been shown to be associated with the risk of developing NAFLD and blood HDL levels.

The importance of the transporter for liver and lung function is demonstrated by the fact that ABCA1 expression in lung tissues ranks second after liver [114]. It is interesting that ABC transporters are differentially expressed in different types of liver cells. Experimental data in mice showed that ABCA1 is expressed mainly in parenchymatous cells and Kupffer cells, whereas another member of the family that is also involved in lipid export and HDL formation, ABCG1, is predominantly in Kupffer cells and endothelium than in parenchymatous cells [115]. These data suggest a different role for ABCA1 and ABCG1 in liver cholesterol export mechanisms. Moreover, a diet with high cholesterol levels results in increased ABCG1 expression in liver parenchyma cells, but these values are still lower than ABCG1 expression levels in endothelial and Kupffer cells, which were not significantly affected by dietary cholesterol [115].

Of the greatest interest is the LXR, which is well known in the pathogenesis of atherosclerosis [116]. Due to its positive role in maintaining lipid balance [117,118,119,120], LXR contributes to the reduction of atherogenesis [121,122,123] and is involved in the regulation of immune response [124,125], possessing anti-inflammatory effects [126]. However, the role of LXR in liver function is not so unambiguous and not only does not correlate with the described positive effects on atherogenesis but, even to the contrary, it demonstrates negative effects, such as hepatic steatosis and hyperlipidemia.

LXR is a ligand-activated transcription factor, presented as two isoforms, LXRα and LXRβ, which are differentially expressed in different organs [127]. LXR is able to bind to cholesterol derivatives and intermediate products of its biosynthesis, as well as polyunsaturated fatty acids and some other compounds [128,129,130,131]. In this case oxysterols act as agonists, while, for example, arachidonic acid, to the contrary, exhibits antagonistic properties [129,130,131].

Thus, LXR is a marker of cellular cholesterol load [132]. LXR activation leads to complex effects on lipid and carbohydrate metabolism, promoting cholesterol export from the liver, stimulating insulin secretion in the pancreas, and increasing its sensitivity in adipose tissue [116].

LXRs show opposite roles in the regulation of de novo lipogenesis in liver and adipose tissue. The absence of LXRs in a mouse experiment stimulates de novo lipogenesis in adipose tissue but suppresses de novo lipogenesis in the liver [133,134]. Thus, despite the supposed positive role of LXR agonists in the prevention of atherosclerosis, their clinical use limits the strong enhancement of lipogenesis in the liver [135].

This is due to the fact that LXR is a direct regulator of genes involved in liver lipogenesis, regulating the expression of SREBP1c and carbohydrate-responsive element-binding protein (ChREBP), SCD1, and Fatty acid synthase (FASN) [136,137]. In an experiment on rats receiving a high-fat diet, a significant increase in LXRα expression was demonstrated [138]. Similar results were obtained in a study of LXRα expression in patients with NAFLD [136]. Nevertheless, in light of increasing knowledge about the involvement of various lipid fractions in the pathogenesis of this disease, it is still too early to unequivocally interpret these results as evidence of LXR involvement in NAFLD progression. It should be considered that the downstream target for LXR is ABCA1, and its function in macrophages contributes to anti-inflammatory action through the removal of excess cellular cholesterol [139]. The use of LXR agonists has also been shown to reduce lipopolysaccharide (LPS)-associated liver inflammation by inhibiting the proinflammatory activity of macrophages through enhanced reverse cholesterol transport via increased expression of the ABCA1 gene.

In addition, the anti-inflammatory effect of LXR may be due to its effect on free fatty acid metabolism in the liver, by increasing SCD1 activity and reducing the concentration of saturated fatty acids in the cells [139,140]. These data indicate that activation of LXR exhibits anti-inflammatory effects in steatosis.

Thus, cholesterol, being the most important cellular component, demonstrates involvement in NAFLD pathogenesis, acting as a link between metabolic disorders and inflammation.

### 3.3. Role of Ceramide

Ceramides belong to the sphingolipid family and are their simplest type. They consist of sphingosine and various fatty acids and participate in the structural organization of the lipid bilayer of plasma membranes [141]. Moreover, the chain length of the fatty acid affects the physical properties of ceramide [142,143,144]. Fatty acids with 16–24 carbon atoms are most often included in the ceramides of mammalian cell membranes because they are the least polar, and the most hydrophobic biophysical properties of ceramide molecules allow them to self-associate by creating microdomains [145,146]. These microdomains have unique biophysical properties and are characterized by high structural rigidity, mechanical stability, and compactness of lipid bilayers and can act as a platform for some signaling molecules [146,147].

Ceramide formation in the exoplasmic leaflet of the cell plasma membrane is associated with the participation of acidic sphingomyelinase and is an important mechanism that affects membrane biophysical properties and their involvement in signal transduction. Additionally, even small changes in ceramide levels can affect the physical properties of membranes [148].

Ceramides may act as a signaling molecule for apoptosis and are associated with the development of many diseases [149]. They are synthesized in various tissues and accumulate in metabolic disorders, dyslipidemia, and inflammation. Elevated ceramide levels are associated with the development of endothelial dysfunction and coronary heart disease and are a prognostic factor for cardiovascular mortality [150]. This allows us to consider circulating levels of ceramides as a marker of cardiovascular diseases [150,151,152] and diabetes type 2 [151,153,154,155,156] and use them as a tool to assess prognosis [150,157]. It is interesting to note that ceramides, which are formed in the liver, are transported to peripheral tissues as part of lipoproteins [157,158,159,160].

The data obtained in recent years have expanded our understanding of the role of ceramides in the pathogenesis of NAFLD. They are involved in many mechanisms of disease pathogenesis as they are involved in fat accumulation, insulin resistance, mitochondrial dysfunction, apoptosis, and fibrosis [58,161].

Ceramides are an important link integrating several lipid metabolic pathways [162]. They enhance fatty acid translocation and uptake because they promote CD36 translocation to the plasma membrane [58,163,164,165,166].

In addition, ceramides also activate signaling pathways that enable TAG synthesis, such as by inducing a major transcriptional regulator of SREBP, which promotes the incorporation of fatty acids into TAGs and facilitates their storage in lipid droplets [58,163]. These processes are related to the energetic characteristics of cells. Under energy-deficient conditions, free fatty acids enter the beta-oxidation pathway, whereas, under energy surplus, these fatty acids act as material for TAG formation, with a view to their subsequent storage as an energy source. However, when TAGs are in excess under conditions of sufficient energy, the fatty acids are diverted into ceramide biosynthesis [162].

Thus, the formation of ceramides *de novo* in the liver is closely related to the metabolic load of the body, such as a diet rich in saturated fats as well as oxidized low-density lipoproteins (LDL) [161,167,168]. In response, ceramides promote preferential use of fatty acids for energy by inhibiting glucose and amino acid absorption [169,170,171]. This mechanism is one of the causes of insulin resistance. Greater fatty acid utilization involving ceramides is also achieved by decreased mitochondrial efficiency, resulting in less energy production in the form of ATP. These mechanisms generally allow for the rapid incorporation of free fatty acids into structural or metabolic processes [154,162,172,173]. Ceramides are important participants in inflammation, participating in the regulation of several pathways [174,175,176]. These and other findings have led to the identification of ceramides as an important integrating factor in an excess lipid load of saturated fatty acids on the body, as well as inflammation and insulin resistance [177].

## 4. Lipid Metabolism Disorders in the Development and Progression of COPD

When initially considered, COPD is not a disease with an obvious lipid metabolism disorder. However, the understanding that the lungs are an organ with a unique lipid biology has significantly broadened the view on the pathogenesis of COPD [178]. Smoking, a major risk factor for COPD, is closely associated with impaired lipid metabolism [179]. Smoking has a significant impact on lung cell function, surfactant composition, and lung lipid structure, including phospholipids, cholesterol, and fatty acids [180,181].

When considering the pathogenesis of COPD, it is important to remember that it is a heterogeneous disease with different pulmonary and extrapulmonary clinical characteristics [182,183,184,185]. Although the concept of COPD phenotypes is still a subject of debate, the two best-known disease phenotypes characterized by emphysema or bronchitis are frequently used in clinical practice [186]. These phenotypes have different pulmonary and extrapulmonary clinical characteristics, including a differential association with the metabolic syndrome. Meanwhile, lower BMI values are described for the emphysematous phenotype [187]. Moreover, known literature data and experimental results confirm the close association of pulmonary emphysema with nutritional deficiencies [188]. It is interesting that cachexia and a decreased BMI predominantly due to muscle mass are prognostically unfavorable characteristics of the natural history of COPD [189]. In contrast, overweight demonstrates the so-called “obesity paradox”, in which patients have better prognostic indicators than low or even normal body weight [190,191].

In addition, it has been shown that airflow parameters such as forced vital capacity (FVC) and FEV1 are associated with the development of metabolic syndrome, inflammation, diabetes, and cardiovascular disease [192,193,194,195,196,197,198,199,200,201,202,203,204,205,206]. These and other data determined a clear relation of COPD with the features of local and systemic metabolism, in which lipid metabolism has an important role.

Disorders of pulmonary lipid metabolism are a well-known pathophysiological characteristic of the processes that are associated with the progression of COPD [180,207]. These changes affect predominantly macrophages, thus providing a close link between impaired pulmonary lipid metabolism and the innate immune response [178]. Numerous articles have been devoted to the study of these connections and an increasing number of them contribute to our better understanding of many of the mechanisms of the innate immune system. Cellular accumulation of cholesterol is an important event that initiates inflammation involving alveolar macrophages. Tobacco smoke has been shown to be involved in reducing the expression and functional activity of ABCA1, which provides reverse cholesterol transport, preventing its accumulation. Cholesterol is considered as a trigger of inflammatory response and decreased phagocytic activity of macrophages. A decrease in the functional activity of ABCA1 can lead to the transformation of macrophages into “foam cells,” which mediate many proinflammatory effects [111].

In experiments, *Abcg1-/-* mice and *Abca1-/-* mice were shown to have abnormal accumulation of cholesterol-overloaded macrophages [208,209,210,211]. An increased number of foamy cells was also recorded in the lungs of smokers [212,213].

Changes in lipid composition of plasma membranes are also considered to be an important pathogenetic mechanism. Cholesterol is known to be a significant structural component of plasma membranes, providing them with necessary biophysical properties, such as viscosity. The unique properties of cholesterol ensure its participation in the lateral organization of lipid bilayer, reducing fluidity and creating the necessary conditions for the optimal localization of membrane proteins. Cholesterol also provides the organization of individual microdomains of the plasma membrane, the so-called lipid rafts, which serve as dynamic platforms for the assembly and functioning of many signaling pathways. Changes in plasma membrane cholesterol content may influence the biophysical properties of plasma membranes and the localization and functional activity of the proteins located therein. It has been suggested that TLR4 activation may be related to the levels of cholesterol in the plasma membrane, which is possible due to specific lipid–protein interactions.

Another important mechanism that is associated with impaired lipid metabolism in the pathogenesis of COPD is the involvement of ceramide [214,215]. Studies have found that ceramide levels are elevated in COPD and demonstrate links to emphysema [215,216]. These findings suggest that ceramide is involved in the pathogenesis of emphysema, which seems compelling given ceramide’s role in apoptosis [214,215,217]. The key events leading to emphysema are thought to be impaired interalveolar septal vascularization, which is associated with apoptosis of endothelial cells and alveolar epithelial cells [218]. Indeed, clinical evidence suggests that plasma levels of sphingomyelins are associated with emphysema, whereas glycosphingolipids are associated with COPD exacerbations [219].

Thus, COPD is characterized by increased local and plasma ceramide concentrations, which, however, do not correlate with the degree of airflow limitation [215]. The increase in ceramide in the systemic bloodstream correlates with plasma activation of acid sphingomyelinase, which is characteristic of smokers [220,221].

It is of great clinical interest to know that the increase in ceramide content in the systemic bloodstream may be associated with the release of microparticles from cells as a result of the damaging effects of cigarette smoke. Microparticles are known to be part of the plasma membrane of cells and are separated from them during cell activation and apoptosis. The ability to generate microparticles is known for various cell types, including endothelial and myeloid cells [222,223,224]. Microparticles are now considered part of a complex mechanism of intercellular interaction and information exchange through the transfer of microRNAs and some proteins, including those thought to have functional activity [225,226]. Microparticles can be taken up by other cells and thus gain a biological link to the original cell. Ceramide levels have been shown to be elevated in smoking and COPD, including by increasing their amounts in pulmonary microparticles [220]. What resultant effect this increase provides is currently not known. However, there is evidence that the uptake of exosomes released upon exposure to tobacco smoke inhibits spleen macrophage efferocytosis [220]. This information is of interest given the negative effect of ceramide on the ability to phagocytose apoptotic cells [227].

Considering that ceramide suppresses amino acid transport and protein synthesis in skeletal muscle cells, an important resulting effect is muscle wasting [169]. Impaired muscle metabolism and muscle wasting is an important characteristic of COPD. Sarcopenia is an independent risk factor for NAFLD in COPD. It is interesting that sarcopenia in patients with COPD is associated with an increased risk of NAFLD and more severe fibrosis independent of lung function and metabolic characteristics [228]. Moreover, worsening histologic severity of NAFLD, especially at the fibrosis stage, is accompanied by decreased lung function [229].

Thus, ceramide plays an important role in the pathogenesis of COPD, given its role in the lipid organization of plasma membranes and the initiation of inflammation.

When considering the role of lipid metabolism in the pathogenesis of COPD, the importance of nuclear LXR receptors should also be emphasized. LXRs play a vital role in cholesterol homeostasis by regulating the transcription of genes involved in macrophage-mediated cholesterol efflux [230].

In patients with COPD, increased LXR expression has been observed in the epithelial cells of the small bronchi and bronchioles, as well as in the alveolar epithelium. At the same time, in alveolar macrophages, no differences in mRNA and LXR protein levels were observed [231]. LXR activation in lungs of patients with COPD may be associated with 25- and 27-hydroxycholesterol, which are endogenous ligands for LXR and whose content is increased in the sputum of patients with COPD [231,232,233]. This corresponds to the increased expression of hydroxylases in alveolar macrophages and pneumocytes responsible for the production of these oxysterols in the lung tissue of patients with COPD [231,232,233].

PPARγ, whose endogenous ligands are lipids, including unsaturated fatty acids and oxidized LDL, can also act as a regulator of LXR transcription [234,235,236,237]. PPAR-γ expression has been shown to be reduced in epithelial cells and myeloid cells of lung tissue in patients with COPD [238,239]. It is interesting that the absence of PPAR in mouse airway epithelial cells was associated with increased susceptibility to cigarette smoke-induced emphysema with excessive macrophage accumulation [240]. Thus, PPARγ is seen as an important negative regulator of cigarette smoke-induced emphysema [239]. In addition, COPD patients have reduced PPAR expression in peripheral skeletal muscles, which may be associated with their dysfunction through impaired regulation of fatty acid homeostasis [241]. These data, as well as information about the anti-inflammatory role of PPARγ, allow researchers to consider PPARγ agonists as a promising therapeutic target [242].

Furthermore, it is known that cigarette smoke can also directly regulate LXR expression in the lungs [231,243,244,245]. This suggests that cholesterol metabolites, fatty acids, and tobacco smoke may influence LXR-dependent cholesterol efflux in lung tissue in COPD [231]. Thus, PPARγ is seen as an important negative regulator of cigarette smoke-induced emphysema [239]. In addition, COPD patients have reduced PPAR expression in peripheral skeletal muscles, which may be associated with their dysfunction through impaired regulation of fatty acid homeostasis [241]. These data, as well as information about the anti-inflammatory role of PPARγ, allow researchers to consider PPARγ agonists as a promising therapeutic target [242].

An important role of LXRs in innate immunity has been associated with macrophages [246]. LXRs demonstrate a complex role in inflammation by inhibiting macrophage apoptosis and negatively regulating proinflammatory cytokine expression induced by LPS and bacteria [246,247,248]. Exposure to LXR agonists in a rat experiment demonstrated a decrease in LPS-induced neutrophilia in the airways [249]. This effect can be explained by the activation of ABCA1 under the influence of LXR signals, which may apparently contribute to the anti-inflammatory (M2) polarization of lung tissue macrophages. The anti-inflammatory effect of LXR activation can also be indirectly judged from data obtained in a mouse experiment that analyzed the effect of suppression of SCD1, the target enzyme for LXR, on the activity of inflammation in the lungs induced by tobacco smoke exposure. According to this study, suppression of SCD1 exacerbated lung inflammatory damage. Another experimental study also demonstrated that exposure to cigarette smoke on alveolar macrophages and pulmonary epithelial cells reduced the expression of the anti-inflammatory LXRβ isoform. However, experimental data using LXR agonists have shown adverse effects of LXR stimulation on host defense against bacterial lung infection. It is suggested that this is due to the complex system of connections between lipid metabolism and innate immunity in which LXR is involved [246,250].

It is interesting that in patients with COPD in the early stages there is a decrease in free fatty acids in blood plasma [251]. Presumably, the high consumption of fatty acids can be explained by the increased need of patients with COPD for high-energy substrates, due to increased metabolic rate due to more intensive respiratory work, as well as the need to maintain inflammation. Acute exposure of distal airway epithelium cells to cigarette smoke has been shown to increase the activity of CPT1A and increase β-oxidation of fatty acids, causing a switch of energy metabolism of cells from glucose, which is the main energy source to lipids [252].

The catabolism of carbohydrates is accompanied by the formation of large amounts of carbon dioxide (CO_2_). The use of fatty acids as an energy substrate contributes to less CO_2_ formation. Against the background of impaired CO_2_ excretion in COPD, getting energy from fatty acids can reduce some negative effects such as muscle dysfunction and shortness of breath [253,254]. Moreover, a diet low in carbohydrates but with the addition of medium chain triglycerides and predominantly monounsaturated fatty acids helps to improve pulmonary function in patients with COPD. At the same time, exposure to cigarette smoke on lung endothelial cells reduces β-oxidation of fatty acids, which leads to increased ceramide synthesis and apoptosis of endothelial cells and, consequently, the risk of emphysema [255].

The composition of free fatty acids in lung tissue shows differences in the stable and in the acute course. In the stable course of the disease, low levels of free alpha-linolenic acid, linoleic acid, and eicosapentaenoic acid were found in the sputum [256]. whereas COPD exacerbation was characterized by higher levels of free arachidonic acid and docosapentaenoic acid, highlighting the contribution of free fatty acids to the mechanisms of COPD [256]. In this case, arachidonic acid acts as a central node, as its metabolites may be differentially involved in inflammation and may be associated with bacterial colonization of the bronchi, which is important for the pathogenesis of COPD and systemic inflammation [257,258,259].

To date, the concept of systemic inflammation in COPD is actively studied and discussed. Among the possible mechanisms of systemic inflammatory response in COPD, we would like to single out the model in which one of the main roles in the process plays a hyperproduction of proinflammatory cytokines such as TNF-1a and IL-1b in blood [260]. In addition to their activating effect in initiating and maintaining systemic inflammation, these cytokines also affect adipose tissue by inducing the production of adipokines such as leptin and adiponectin, which are extremely biologically active and can influence fat metabolism and energy metabolism in general. Currently, the effect of adipokines is considered to be an important link in the pathogenesis of NAFLD, the development of insulin resistance, and metabolic syndrome [261,262,263].

Thus, systemic inflammation in COPD is an important mechanism that may affect the function of other organs and tissues. The systemic inflammatory process, which is part of the pathogenesis of COPD, may lead to the development or exacerbation of comorbidities such as type 2 diabetes and metabolic syndrome [264,265,266].

Another important factor that is associated with the development of extrapulmonary clinical heterogeneity of COPD is the resultant effects on the organs of hypoxia due to impaired pulmonary function. Hypoxia activates the hypoxia-inducible factor (HIF) stabilization-related signaling pathway. HIF family of transcription factors are heterodimers that consist of an alpha-subunit (HIF1a, HIF2a, and HIF3a) and a beta-subunit. It is known that HIF-1α and HIF-2α can have different transcriptional targets and can be differently involved in disease pathogenesis [267].

Research of the HIF-1α isoform role in the pathogenesis of COPD has shown that high HIF-1α expression may be associated with decreased lung function and quality of life, which contribute to disease progression [268]. Increased expression of HIF-1α may also be associated with bacterial lung colonization and infectious exacerbations because HIF-1α can regulate the platelet-activating factor receptor (PAFR) on the airway epithelium surface, which is used by PAFR-dependent bacteria (Streptococcus pneumoniae, Haemophilus influenzae, and Pseudomonas aeruginosa) [269].

The development of NAFLD is closely related to hypoxia [270]. Experimental data showed that a high-fat diet resulted in severe hypoxia and triglyceride accumulation in the liver of mice [271].

Research suggests that HIF-2α activation in hepatocytes is a key feature of NAFLD and contributes significantly to disease progression [272]. It was shown that steatotic hepatocytes, in comparison with normal hepatocytes, exhibited a higher sensitivity to hypoxia, demonstrating lipid accumulation. This is associated with the fact that hyperexpression of HIF-2α, which develops under hypoxia, suppresses PPARa in the liver and, consequently, aggravates the course of NAFLD [273]. Hypoxia is characterized by inhibition of genes related to β-oxidation, while, on the contrary, lipogenesis genes, including LXRa, FAS, and SCD1, are activated [273]. In turn, HIF1-α plays an important role in the development of liver fibrosis in NAFLD [274,275]. HIF-1α was found to be significantly elevated in fibrotic liver tissues and activated HSCs; blocking HIF-1α expression inhibited HSCs’ proliferation and activation [276,277,278].

It is interesting that the role of the HIF-2α isoform in the pathogenesis of COPD turned out to be opposite: In an experiment on mice, a more severe emphysema developed when HIF-2α was deleted, whereas a protective effect was observed when HIF-2α was hyperexpressed. Thus, HIF-2α may be a key endogenous factor preventing the development of emphysema in COPD. HIF-2α can maintain alveolar architecture by promoting vascular survival [279]. HIF-2α protein levels in the lung tissue of COPD patients are decreased, which is associated with exposure to tobacco smoke [280].

Thus, hypoxia is an important factor contributing to the integration of several inflammatory and metabolic pathways that may be involved in the pathogenesis of COPD and NAFLD.

## 5. Conclusions

The results of this review suggest that NAFLD and COPD have multiple mechanisms of development, in which impaired lipid metabolism is central.

**Figure 3:**
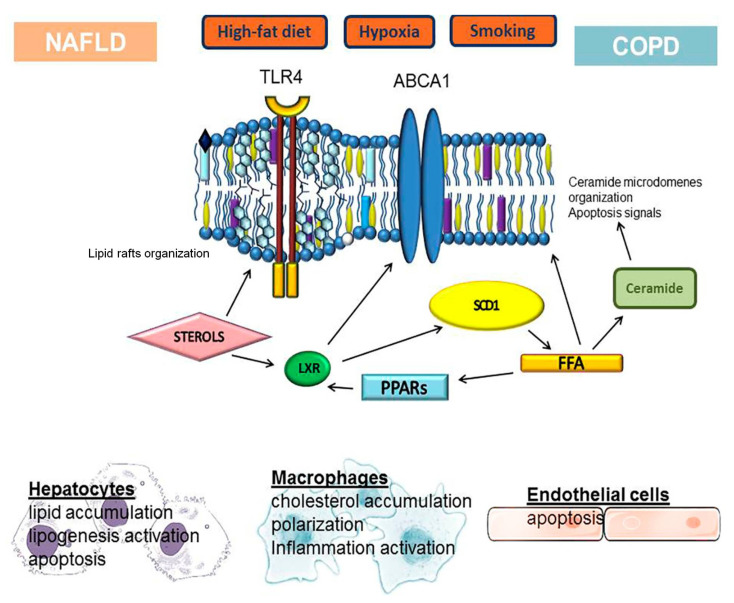
Schematic demonstrating the key initiating, intermediate, and resulting factors associated with cellular disorders of lipid metabolism in the pathogenesis of NAFLD and COPD.

Moreover, although the etiopathogenesis of these two diseases significantly differs, lipid metabolism disorder may be a key link in the comorbid course of NAFLD and COPD (Figure 3). However, some cellular mechanisms underlying the development of both diseases show certain similarities. At present, there is insufficient research data to speak unequivocally about the reciprocal influence of NAFLD on COPD and vice versa.

TAG accumulation in hepatocytes in NAFLD has a complex nature, which is associated with the participation of many exogenous and endogenous factors. Various lipids are involved in these processes, including cholesterol associated with inflammation, due to its role in providing the structure of plasma membranes and ensuring the functioning of many signaling pathways. Disrupted metabolism of fatty acids is one of the central links in the pathogenesis of NAFLD and COPD due to their involvement not only as an energy substrate but also to their structural function in cells and to their connection with inflammation. Ceramides, due to their role as a link integrating several pathways of lipid metabolism and their participation as a signaling molecule, are also associated with TAG synthesis and are involved in the formation of the clinically heterogeneous course of COPD. Analysis of these processes also indicates a close relationship between lipid metabolism and inflammation.

Nuclear receptors play a prominent role in the pathogenesis of both diseases. It is known that the activation of nuclear receptors plays a prominent role in the pathogenesis of both diseases, the LXRs’ family being of particular importance among them. Activation of this nuclear receptor induces ABCA1-mediated elimination of excess cholesterol from cells and reduces lipotoxicity by increasing SCD1 activity and reducing the concentration of saturated fatty acids in cells. Additionally, LXR-mediated processes stimulate M2 anti-inflammatory differentiation of macrophages in the liver and lungs. Thus, LXR activation in cells may have a protective effect aimed at reducing the intensity of inflammation and, in both cases, can serve as a marker of cellular lipid overload. Nevertheless, in spite of the general protective role, hyperexpression of LXR in the liver is accompanied by excessive accumulation of lipids in the liver, through stimulation of lipogenesis de novo by the mechanism of activation of SREBPs and ChREBP family proteins, which makes the role of this cellular receptor in the pathogenesis of NAFLD ambiguous. Meanwhile, in lung macrophages of COPD patients, cholesterol accumulation associated with decreased expression and functional activity of ABCA1 may be associated with inflammation.

Members of the nuclear receptor family PPAR, acting as sensors of lipid molecules such as fatty acids, play a key role in the regulation of metabolic homeostasis and are closely related to the pathogenesis of NAFLD and may also be involved in the pulmonary clinical heterogeneity of COPD.

The existing epidemiological and clinical data, as well as numerous experimental works confirming the opinion that NAFLD and COPD, being widespread noncommunicable diseases with systemic lifestyle components, have common pathogenetic mechanisms, which explain the high frequency of a comorbid course that is primarily associated with metabolic disorders and its cross-linkages with systemic inflammation in COPD, which can also contribute to NAFLD progression.

Our analysis highlights the need for a better understanding of the multifaceted role of lipid metabolism and its disorders as a link in a complex chain of processes underlying the development of long-term diseases such as NAFLD and COPD. The pathogenetic mechanisms of these diseases may overlap throughout their natural history, and lipids are an integral part of such overlaps.

## Data Availability

Not applicable.
